# Prevalence and distribution of livestock schistosomiasis and fascioliasis in Côte d’Ivoire: results from a cross-sectional survey

**DOI:** 10.1186/s12917-020-02667-y

**Published:** 2020-11-17

**Authors:** Jules N. Kouadio, Jennifer Giovanoli Evack, Louise Y. Achi, Dominik Fritsche, Mamadou Ouattara, Kigbafori D. Silué, Bassirou Bonfoh, Jan Hattendorf, Jürg Utzinger, Jakob Zinsstag, Oliver Balmer, Eliézer K. N’Goran

**Affiliations:** 1 Unité de Formation et de Recherche Biosciences, Université Félix Houphouët-Boigny, 22 BP 770 Abidjan 22, Abidjan, Côte d’Ivoire; 2 Centre Suisse de Recherches Scientifiques en Côte d’Ivoire, 01 BP 1303 Abidjan 01, Abidjan, Côte d’Ivoire; 3 Swiss Tropical and Public Health Institute, P.O. Box CH-4002 Basel, Switzerland; 4 University of Basel, P.O. Box CH-4001 Basel, Switzerland; 5 Ecole de Spécialisation en Elevage et des Métiers de la Viande de Bingerville, BP 58 Bingerville, Abidjan, Côte d’Ivoire; 6 University of Zurich, Ramistrasse 71, P.O. Box CH-8006 Zurich, Switzerland

**Keywords:** Côte d’Ivoire, Cross-sectional survey, Epidemiology, *Fasciola*, Livestock, *Schistosoma*

## Abstract

**Background:**

*Schistosoma* and *Fasciola* are zoonotic parasites of public health and veterinary importance. However, while the epidemiology of schistosomiasis in humans is well studied, little is known about fascioliasis and schistosomiasis in livestock in Côte d’Ivoire. This study aimed to determine the prevalence and the distribution of livestock schistosomiasis and fascioliasis across Côte d’Ivoire.

In 2018, we conducted a cross-sectional survey in abattoirs and farms in 13 departments of Côte d’Ivoire. In abattoirs, the mesenteric veins and livers of slaughtered cattle, sheep and goats were examined for adult *Schistosoma* and *Fasciola* flukes. Faeces from live cattle, goats and sheep were collected and examined for *Schistosoma* and *Fasciola* eggs using a sedimentation technique.

**Results:**

A total of 386 cattle, 174 goats and 151 sheep from abattoirs and 435 cattle, 22 goats and 176 sheep from farms were sampled. The observed prevalence of schistosomiasis was higher in slaughtered animals. Fascioliasis was more prevalent in farm animals. The prevalence of schistosomiasis in slaughtered cattle varied between 5.9% (95% confidence interval (CI): 0.7–19.7%) and 53.3% (95% CI: 37.9–68.3%) with the highest prevalence observed in Ouangolodougou in the North. Cattle from farms had a relatively low prevalence of schistosomiasis, with the highest prevalence found in Ouangolodougou (2.4%, 95% CI: 0.7–6.1%). The prevalence of fascioliasis varied considerably from one department to another, ranging from nil (95% CI: 0.0–18.5%) to 50.8% (95% CI: 43.4–58.2%), with the highest prevalence found in farm cattle in Dikodougou in the North. Sheep and goats had a lower prevalence of schistosomiasis and fascioliasis than cattle. In slaughtered animals, cattle aged 4 years and older were at highest risk for schistosomiasis (odds ratio (OR): 2.4; 95% CI: 1.0–5.6) and fascioliasis (OR: 2.1; 95% CI: 1.1–3.9). In farm animals, male cattle had higher odds of being infected with *Schistosoma* (OR: 4.3; 95% CI: 0.7–26.9) than females.

**Conclusions:**

Our study confirms that schistosomiasis and fascioliasis are endemic in livestock across Côte d’Ivoire. A strategic control programme should be considered, especially for cattle, including providing drinking water in troughs to reduce faecal contamination of water sources by cattle.

**Supplementary Information:**

The online version contains supplementary material available at 10.1186/s12917-020-02667-y.

## Background

Schistosomiasis and fascioliasis are zoonotic diseases with a complex transmission cycle involving aquatic snails as intermediate hosts and mammalian definitive hosts [1, 2]. Schistosomiasis and fascioliasis are both caused by trematodes; the former by the genus *Schistosoma*, the latter by the genus *Fasciola* [3, 4]. Schistosomiasis and fascioliasis are widespread in tropical and subtropical regions of the world where climatic, ecological and hygienic conditions favour their transmission [3, 5]. Besides their considerable public health burden [6], schistosomiasis and fascioliasis are responsible for economic loss in livestock, mainly through reduced fertility and productivity, liver condemnation, stunted growth and premature death [7–9].

Schistosomiasis also exists in wild and domestic animals and a wide range of mammals are susceptible to schistosome infection, including buffaloes, camels, cattle, goats, horses, pigs and sheep [10–12]. *Schistosoma* species that affect mammals include *Schistosoma bovis*, *S. curassoni*, *S. hippopotami,, S. indicum*, *S. intercalatum*, *S. mattheei*, *S. nasalis*, *S. rohhaini* and *S. spindale* [13]*.* In many countries, *S. bovis* is one of the main species of veterinary and zoonotic importance [8, 14, 15]. In West Africa, *S. bovis* and *S. curassoni* are responsible for schistosomiasis in cattle and small ruminants (goat and sheep), respectively [16, 17]. The prevalence of bovine schistosomiasis varies greatly from 1.2% in Nigeria [18] to 21.7% in Ghana [19]. In Côte d’Ivoire, human schistosomiasis has been the subject of numerous epidemiological studies [20–22]. In contrast, livestock schistosomiasis, has received little attention [23], despite the economic importance of livestock in the country. Prevalence of livestock schistosomiasis has been reported at 35% in the northern part of the country [23]. Of note, recent studies have found that *S. bovis* is involved in the hybridisation of schistosomes in humans in Côte d’Ivoire [24, 25].

Fascioliasis is a chronic disease with a global distribution that mainly occurs in domestic ruminants [26, 27]. There are two *Fasciola* species; namely *Fasciola hepatica* and *F. gigantica*. The former is a cosmopolitan species adapted to temperate areas, while *F. gigantica* is responsible for fascioliasis in tropical and subtropical regions of Africa and Asia [28–30]. In areas where both species coexist, *F. hepatica* x *F. gigantica* hybrids have been found [31–33]. Recently, the presence of hybrids has also been confirmed in sub-Saharan Africa [34]. Fascioliasis is endemic in West Africa with the predominant species being *F. gigantica* [35, 36]. The disease has been reported in cattle with prevalence rates of 6.4–24.8% in Benin [37], 51.1% in Ghana [19], 7–50% in Mali [38] and 28–75% in Nigeria [18, 39–42]. Fascioliasis was also noted in North Côte d’Ivoire in 2003, with a prevalence of *F. gigantica* in cattle of 4% [23], however very little knowledge on the disease situation has been gained since then. Liver condemnation due to fascioliasis is frequent in abattoirs across the country.

The purpose of this study was to provide an overview of the epidemiology of schistosomiasis and fascioliasis in livestock by investigating the prevalence and the distribution of these diseases across Côte d’Ivoire. We designed a cross-sectional survey in abattoirs to collect adult flukes from slaughtered animals and surveyed animals on farms to identify parasite eggs in their faeces.

## Results

### Characteristics of the animal populations

A total of 711 slaughtered animals (386 cattle, 174 goats and 151 sheep) from abattoirs and 933 farm animals (735 cattle, 22 goats and 176 sheep) were sampled. Most animals were females, consisting of at least 61.4% in each group. Cattle breeds included “Taurin”, “Zébu” and “Taurin x Zébu”. “Zébu” were the most commonly slaughtered cattle (42.5%), while “Taurin x Zébu” were the most common breed found on the farms (79.9% of live cattle). Most of the cattle (79.6%) were at least 4 years old, both on farms and in abattoirs. As in cattle, more than 60% of the goats and sheep sampled were females on farms and in abattoirs. Goat breeds consisted of “Naine” and “Sahelien”. The local breed “Naine” was the most common, at 98.9% and 100% of slaughtered and live goats, respectively. Sheep breeds included “Djallonké”, “Sahelien” and “Djallonké x Sahelien”. As with goats, the local breed “Djallonké” was the most common breed at 98.7% and 89.2% in abattoirs and on farms, respectively.

### Prevalence of *Schistosoma* and *Fasciola*

#### Prevalence of *Schistosoma* and *Fasciola* infections in slaughtered animals

Post-mortem examination of livers and small intestines in slaughterhouses revealed the presence of *Schistosoma* spp. and *F. gigantica* in 12.8% and 11.3%, respectively, of all slaughtered animals (cattle, goats and sheep). The mean prevalence of *Schistosoma* and *Fasciola* flukes in cattle across all departments was 22.5% and 19.7%, respectively (Table 1). Goats and sheep were considerably less often infected than cattle (Table 1). In goats, no *Fasciola* flukes were found, whereas *Schistosoma* flukes were found in 1.2% of the animals (Table 1). In sheep, *Schistosoma* and *Fasciola* flukes were found at a prevalence of 1.3% and 2.7%, respectively (Table 1). The highest prevalence of *S. bovis* and *F. gigantica* were found in slaughtered cattle, 53.3% and 40.6%, respectively.

**Table 1:** Prevalence of *Schistosoma* and *Fasciola* infection in slaughtered cattle (*n* = 386), goats (*n* = 174) and sheep (*n* = 151) in 11 departments of Côte d’Ivoire in a cross-sectional survey conducted in March 2018

Department	Cattle					Goats					Sheep				
	N	*Schistosoma bovis*		*Fasciola gigantica*		N	*Schistosoma* spp.		*Fasciola gigantica*		N	*Schistosoma* spp.		*Fasciola gigantica*	
		n_pos_	% (95% CI)	n_pos_	% (95% CI)		n_pos_	% (95% CI)	n_pos_	% (95% CI)		n_pos_	% (95% CI)	n_pos_	% (95% CI)
Ouangolodougou	45	24	53.3 (37.9–68.3)	7	15.6 (6.5–29.5)	9	0	0.0 (0.0–33.6)	0	0.0 (0.0–0.3)	6	0	0.0 (0.0–45.9)	0	0.0 (0.0–45.9)
Ferkessédougou	57	14	24.6 (14.1–37.8)	6	10.5 (4.0-21.5)	29	2	6.9 (0.8–22.8)	0	0.0 (0.0–0.1)	14	1	7.1 (0.2–33.9)	0	0.0 (0.0–23.2)
Korhogo	52	18	34.6 (22.0–49.1)	14	26.9 (15.6–41.0)	36	0	0.0 (0.0–9.7)	0	0.0 (0.0–0.1)	36	0	0.0 (0.0–9.7)	1	2.8 (0.1–14.5)
Niakaramadougou	18	2	11.1 (1.4–34.7)	0	0.0 (0.0–18.5)	25	0	0.0 (0.0–13.7)	0	0.0 (0.0–0.1)	6	0	0.0 (0.0–45.9)	0	0.0 (0.0–45.9)
Katiola	32	3	9.4 (2.0–25.0)	13	40.6 (23.7–59.4)	6	0	0.0 (0.0–45.9)	0	0.0 (0.0–0.5)	–	–	–	–	–
Bouaké	50	6	12.0 (4.5–24.3)	10	20.0 (10.0–33.7)	7	0	0.0 (0.0–41.0)	0	0.0 (0.0–0.4)	9	0	0.0 (0.0–33.6)	0	0.0 (0.0–33.6)
Yamoussoukro	34	6	17.6 (6.8–34.5)	8	23.5 (10.7–41.2)	–	–	–	–	–	–	–	–	–	–
Toumodi	16	2	12.5 (1.6–38.3)	0	0.0 (0.0–20.6)	–	–	–	–	–	–	–	–	–	–
Agboville	27	5	18.5 (6.3–38.1)	4	14.8 (4.2–33.7)	20	0	0.0 (0.0–16.8)	0	0.0 (0.0–16.8)	42	0	0.0 (0.0–8.4)	0	0.0 (0.0–8.4)
Sikensi	21	5	23.8 (8.2–47.2)	6	28.6 (11.3–52.2)	2	0	0.0 (0.0–84.2)	0	0.0 (0.0–84.2)	13	1	7.7 (0.2–36.0)	1	7.7 (0.2–36.0)
Duekoué	34	2	5.9 (0.7–19.7)	8	23.5 (10.8–41.2)	40	0	0.0 (0.0–8.8)	0	0.0 (0.0–8.8)	25	0	0.0 (0.0–13.7)	2	8.0 (1.0–26.0)
Total	386	87	22.5 (18.5–27.0)	76	19.7 (15.8–24.0)	174	2	1.2 (0.1–4.1)	0	0.0 (0.0–2.1)	151	2	1.3 (0.2–4.7)	4	2.7 (0.7–6.6)

*N* Number of investigated animals per department, *n*_*pos*_ Number of infected animals, *CI* Confidence interval

The association between parasitic infection and sex, age and breed of slaughtered cattle are summarised in Table 2. Cattle aged 4 years and older showed the highest odds of *S. bovis* infection (multiple OR: 2.4; 95% CI: 1.0–5.6). There was no significant association with sex (multiple OR: 1.1; 95% CI: 0.6–1.8). The breeds “Taurin” (multiple OR: 0.9; 95% CI: 0.3–2.8) and “Zébu” (multiple OR: 0.6; 95% CI: 0.2–1.8) had lower odds of infection than “Taurin x Zébu”.

**Table 2:** Multiple logistic GEE model analysis of variables associated with *Schistosoma bovis* and *Fasciola gigantica* infection among slaughtered cattle, adjusted for potential correlation within slaughterhouse in a cross-sectional survey conducted in Côte d’Ivoire in March 2018. Note that 82 animals from Agboville, Sikensi and Duekoué were not included in the analysis because of missing data on age

Trait	N	*Schistosoma bovis*			*Fasciola gigantica*		
		Infected n_pos_ (%)	Multiple OR	95% CI	Infected n_pos_ (%)	Multiple OR	95% CI
Sex							
Female	192	49 (25.5)			37 (19.3)		
Male	112	26 (23.2)	1.1	0.6–1.8	21 (18.8)	1.0	0.6–1.6
Age (years)							
1–3	62	9 (14.5)			7 (11.3)		
≥ 4	242	66 (27.3)	2.4	1.0–5.6	51 (21.1)	2.1	1.1–3.9
Breed							
Taurin x Zébu	107	31 (29.0)			26 (24.3)		
Taurin	77	20 (26.0)	0.9	0.3–2.8	8 (10.4)	0.4	0.2–0.9
Zébu	120	24 (20.0)	0.6	0.2–1.8	24 (20.0)	0.7	0.5–1.1

*N* Investigated animals, *n*_*pos*_ Number of infected animals, *CI* Confidence interval, *OR* Odds ratio

In regards to fascioliasis, cattle aged 4 years and older had the highest odds of infection (multiple OR: 2.1; 95% CI: 1.1–3.9). Sex showed no association with fascioliasis (1.0; 95% CI: 0.6–1.6). The breeds “Taurin” (multiple OR: 0.4; 95% CI: 0.2–0.9) and “Zébu” (multiple OR: 0.7; 95% CI: 0.5–1.1) had lower odds of infection compared to “Taurin x Zébu”.

#### Prevalence of *Schistosoma* and *Fasciola* infections in farm animals

Farm animals were found infected with *Schistosoma* spp. and *F. gigantica*, as determined by parasite eggs in faecal samples. In cattle, the overall prevalence of *S. bovis* and *F. gigantica* was 0.7% and 29.5%, respectively (Table 3). The prevalence of *Schistosoma* spp. was higher in goats than in sheep, while the contrary was observed for *F. gigantica*; but no significant difference was observed. The highest prevalence of *Schistosoma* (13.6%) and *Fasciola* (50.8%) were found in goats and cattle, respectively.

**Table 3:** Prevalence of *Schistosoma* and *Fasciola* in farm cattle (*n* = 735), goats (*n* = 22) and sheep (*n* = 176) in four departments of the northern part of Côte d’Ivoire in a cross-sectional survey carried out in August 2018

Department	Cattle					Goats					Sheep				
	N	*Schistosoma bovis*		*Fasciola gigantica*		N	*Schistosoma* spp.		*Fasciola gigantica*		N	*Schistosoma* spp.		*Fasciola gigantica*	
		n_pos_	% (95% CI)	n_pos_	% (95% CI)		n_pos_	% (95% CI)	n_pos_	% (95% CI)		n_pos_	% (95% CI)	n_pos_	% (95% CI)
Ouangolodougou	166	4	2.4 (0.7–6.1)	37	22.3 (16.2–29.4)	–	–	–	–	–	6	0	0.0 (0.0–45.9)	0	0.0 (0.0–45.9)
Ferkessédougou	199	1	0.5 (0.0–2.8)	33	16.6 (11.7–22.5)	22	3	13.6 (2.9–34.9)	0	0.0 (0.0–15.4)	60	0	0.0 (0.0–6.0)	5	8.3 (2.8–18.4)
Sinématiali	185	0	0.0 (0.0–2.0)	53	28.6 (22.3–35.7)	–	–	–	–	–	90	1	1.1 (0.0–6.0)	11	12.2 (6.3–20.8)
Dikodougou	185	0	0.0 (0.0–2.0)	94	50.8 (43.4 58.2)	–	–	–	–	–	20	0	0.0 (0.0–16.8)	0	0.0 (0.0–16.8)
Total	735	5	0.7 (0.2–1.6)	217	29.5 (26.3–33.0)	22	3	13.6 (2.9–34.9)	0	0.0 (0.0–15.4)	176	1	0.6 (0.0–3.1)	16	9.1 (5.3–14.3)

*N* Number of animals per department, *n*_*pos*_ Number of infected animals, *CI* Confidence interval

The association between parasitic infections and sex, age and breed of farm cattle are shown in Table 4. Males had higher odds of being infected with *S. bovis* (multiple OR: 4.3; 95% CI: 0.7–26.9). Cattle aged 4 years and older had lower odds of *Schistosoma* infection compared to cattle younger than 4 years (multiple OR: 0.6; 95% CI: 0.2–2.3), but these associations lacked statistical significance.

**Table 4:** Multiple logistic GEE model analysis of variables associated with *Schistosoma bovis* and *Fasciola gigantica* infection among farm cattle, adjusted for potential correlation within farms in a cross-sectional survey carried out in Côte d’Ivoire in August 2018

Trait	N	*Schistosoma bovis*			*Fasciola gigantica*		
		Infected n_pos_ (%)	Multiple OR	95% CI	Infected n_pos_ (%)	Multiple OR	95% CI
Sex							
Female	563	2 (0.4)			173 (30.7)		
Male	172	3 (1.7)	4.3	0.7–26.9	44 (25.6)	1.1	0.8–1.7
Age (years)							
1–3	150	2 (1.3)			33 (22.0)		
≥ 4	585	3 (0.5)	0.6	0.2–2.3	184 (31.5)	1.7	1.0–2.9
Breed							
Taurin x Zébu	587	4 (0.7)			183 (31.2)		
Taurin	37	0 (0.0)	ND	ND	10 (27.0)	0.8	0.3–2.5
Zébu	111	1 (0.9)	ND	ND	24 (21.6)	0.6	0.3–1.0

*N* Investigated animals, *n*_*pos*_ Number of infected animals, *CI* Confidence interval, *ND* Not determined because of low number of infected individuals in several categories, *OR* Odds ratio

For fascioliasis, sex showed no strong association (multiple OR: 1.1; 95% CI: 0.8–1.7). Cattle aged 4 years and older had higher odds of infection compared to their younger counterparts (multiple OR: 1.7; 95% CI: 1.0–2.9). The breeds “Taurin” (multiple OR: 0.8; 95% CI: 0.3–2.5) and “Zébu” (multiple OR: 0.6; 95% CI: 0.3–1.0) had lower odds of infection compared to the “Taurin x Zébu”, but the difference lacked statistical significance.

### Spatial distribution of *Schistosoma* and *Fasciola* infection in livestock

**Fig. 1:**
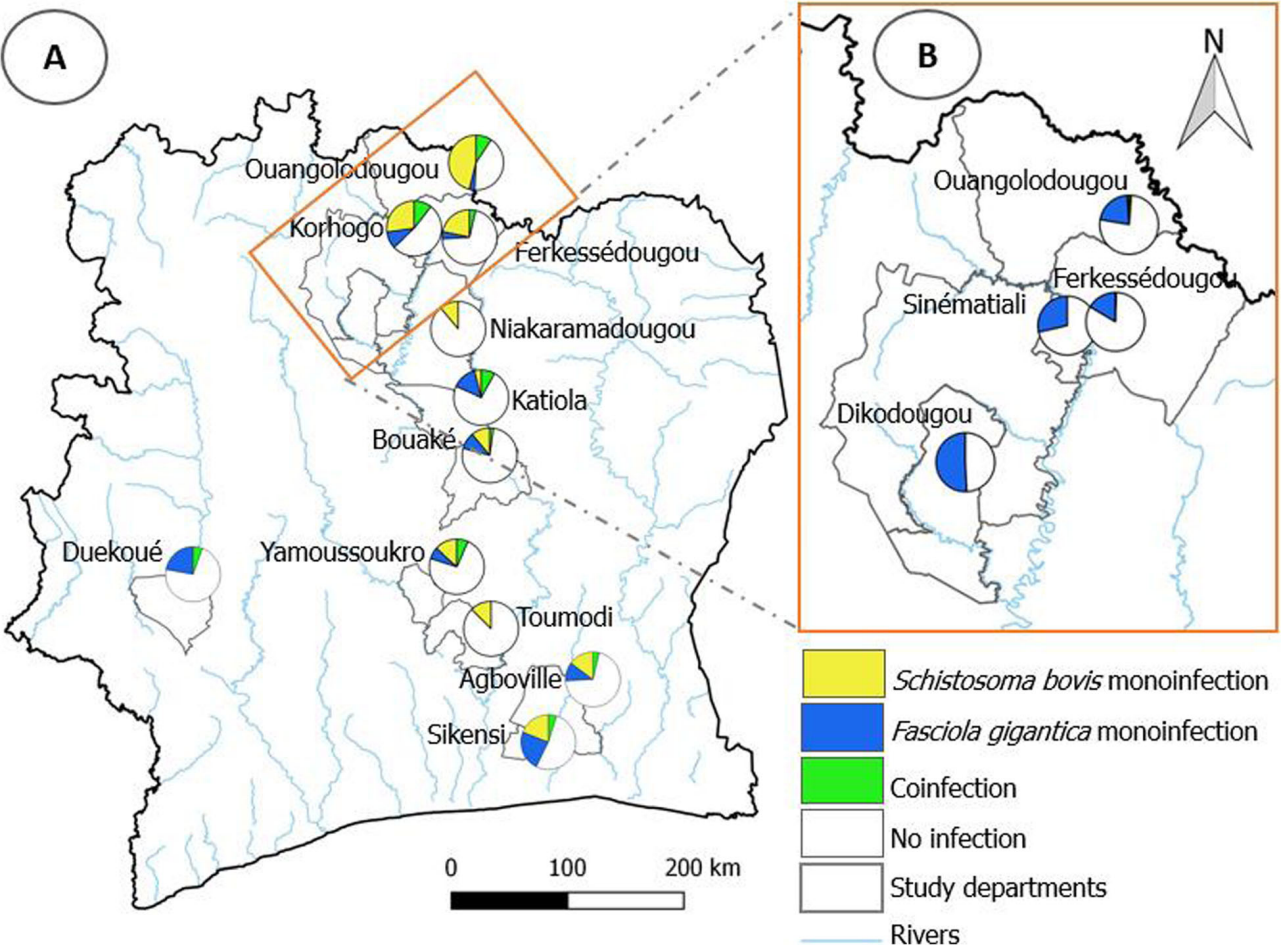
Map of Côte d’Ivoire showing the proportions of animals found infected with either *Schistosoma* or *Fasciola* or both fluke species concurrently in each department, stratified by surveys conducted in abattoirs (**a**) and on farms (**b**). Software QGIS version 2.16.0 ‘Nødebo’ (QGIS Development Team) [43], was used to make the map

The prevalence of schistosomiasis and fascioliasis varied by department and host species, with the highest prevalence observed in the northern part of Côte d’Ivoire (Fig. 1). As shown in Table 1, the highest prevalence of schistosomiasis in cattle sampled in slaughterhouses was found in Ouangolodougou (53.3%, 95% CI: 37.9–68.3%), while the highest prevalence of fascioliasis was observed in Katiola (40.6%, 95% CI: 23.7–59.4%). The lowest prevalence of schistosomiasis in slaughtered cattle was observed in Duekoué (5.9%, 95% CI: 0.7–19.7%). Fascioliasis was absent in slaughtered cattle in Niakaramadougou and Toumodi.

In farm cattle, *F. gigantica* was considerably more prevalent than *S. bovis* (Fig. 3b). As summarized in Table 3, the highest prevalence of *F. gigantica* in farm cattle was observed in Dikodougou (50.8%), whilst the highest prevalence of *S. bovis* was found in Ouangolodougou (2.4%).

In slaughtered sheep, *Schistosoma* spp. infection was present in Ferkessédougou and Sikensi with respective prevalences of 7.1% (95% CI: 0.2–33.9%) and 7.7% (95% CI: 0.2–36.0%). Slaughtered goats were also found infected with *Schistosoma* spp. in Ferkessédougou (6.9%; 95% CI: 0.0–9.7%). In Korhogo, sheep were infected with *F. gigantica* (2.8%; 95% CI: 0.1–14.5%), but not with *Schistosoma* and goats were not infected with either parasite. In slaughtered goats and sheep, the highest prevalence of *Fasciola* infection was recorded in sheep in Duekoué (8.0%; 95% CI: 1.0–26.0%). On farms, *Schistosoma* spp*.* were detected in sheep in Sinématiali (1.1%; 95% CI: 0.0–6.0%) and goats in Ferkessédougou (13.6%; 95% CI: 2.9–34.9%) (Table 3). Fascioliasis was found in farm sheep with a prevalence of 8.3% (95% CI: 2.8–18.4%) and 12.2% (95% CI: 6.3–20.8%) in Ferkessédougou and Sinématiali, respectively (Table 3).

## Discussion

The prevalence and distribution of trematodes belonging to the genera *Schistosoma* and *Fasciola* in slaughtered and live livestock were assessed in the savannah (Centre and North) and forest (South and West) areas of Côte d’Ivoire. The results showed that schistosomiasis and fascioliasis are endemic in Côte d’Ivoire. *Schistosoma* and *Fasciola* infection were found at all the investigated sites. Cattle were infected more often with *Schistosoma* spp. and *F. gigantica* than goats and sheep, both in slaughterhouses and on farms. Sex was not associated with parasitic infections in slaughtered animals. However, in farm cattle, males were infected more often with *Schistosoma* than females and the spatial distribution of infections showed that the highest prevalence was found in the northern part of the country.

The distribution of *Schistosoma* and *Fasciola* is governed by the presence of multipurpose dams and other freshwater bodies (e.g. rivers) and animal-water contact sites (for schistosomiasis) and consumption of aquatic plants (for fascioliasis). The river networks running North to South is of particular relevance, as it provides habitats for intermediate snail hosts. In addition, the livestock system itself may play an essential role in maintaining the transmission of schistosomiasis. In traditional livestock-systems, animals graze and drink water near and from rivers and dams, depositing faeces containing the parasite eggs, perpetuating the life cycle. A study carried out in the southern highlands of Tanzania demonstrated that livestock management systems influence the epidemiology of trematode infection. Indeed, it was found that the prevalence of trematodes was high in areas where traditional systems were practiced, moderate in large-scale dairy systems and lowest in small-scale dairy systems [44]. In Mali, another study showed that factors such as climatic conditions, presence of rivers and lakes, and livestock management practices influence the prevalence of trematode infections [38].

The prevalence of schistosomiasis and fascioliasis in goats and sheep was low compared to cattle. Cattle graze away from the villages and consume water from rivers and dams. Goats and sheep, in contrast, often graze around the villages and consume water that is provided by farmers. This likely reduces the contact with infested freshwater and reduces the risk of *Schistosoma* infection in goats and sheep. Our findings are in line with a previous study in the south-eastern Lake Chad area that reported a higher prevalence of adult liver flukes in cattle than sheep and goats [45]. Frequent grazing of cattle near the lake emerged as the key risk factor. In Iran, another study also reported a lower prevalence of *Fasciola* in goats and sheep compared to cattle. This observation was explained by the fact that goats consume leaves and heaths in elevated areas, sheep graze on open land, while cattle pasture near the springs and streams, and hence, cattle are at a higher risk of exposure to snail-infested freshwater [46].

The two trematode infections appear to be more prevalent in Côte d’Ivoire than elsewhere in sub-Saharan Africa. Indeed, the prevalence of 53.3% of *Schistosoma* is more than 2-fold higher than that recorded in South Ghana (21.7%) [19]. The prevalence of *Fasciola* (50.8%) in the current study is higher than previously recorded in the northern part of Côte d’Ivoire (4%) [23]. Our prevalence estimate is also higher compared to research conducted in Ethiopia (31.3%) [47]. However, other studies also showed prevalences above 50%; for instance in Chad (68%) [45], Ghana (51.1%) [19] and Zambia (53.9%) [48]. These high rates in Côte d’Ivoire may be due to the extensive farming systems with little or no veterinary input. The lack of a control programme against trematodes leads to livestock owners medicating their animals with commonly used anthelminthic drugs such as albendazole and nitroxynil without a prescription from a veterinary. Triclabendazole [49], the drug of choice against *Fasciola* is not available for use in Côte d’Ivoire. Praziquantel, which is highly effective against all visceral bovine schistosomiasis [50], is also not commercially available for livestock in the country. These factors likely contribute to the endemicity of livestock schistosomiasis and fascioliasis.

*Schistosoma* infection was particularly high in male cattle on farms. A prior study from Nigeria showed that the prevalence of *Fasciola* was higher in male cattle than in females [51]. In that study, the authors speculated that the high infection rate in male cattle could be attributed to the fact that the males were more often slaughtered for consumption, while the females were left for milk production and breeding. This may have led the herdsmen to ensure that the females graze on clean pasture and drink clean water [51].

In slaughtered animals, sex was not associated with *Schistosoma* and *Fasciola* infection. In contrast to the aforementioned Nigerian abattoir survey [51], the prevalence of *Schistosoma* and *Fasciola* infection in farm cattle was higher in males than in females. Results concerning this issue are thus not consistent, as there are studies that report higher prevalence in male cattle [13, 52] and others that report higher prevalence in females [53, 54].

Cattle aged 4 years and older were infected more often by *S. bovis* and *F. gigantica* than their younger counterparts in slaughterhouses. This finding might be explained by the fact that farmers predominantly butcher older animals or animals that are ill to avoid the death of the animal on the farm, which would result in economic losses. It is conceivable that older animals are in contact with contaminated water for longer periods than younger animals that are kept on the farm for breeding. This is in agreement with studies that reported similar findings in Nigeria [16, 18] and Tanzania [55, 56]. Others studies from western Ethiopia [47] and in the Philippines [57] showed a statistically significant association of the age of the cattle with the prevalence of fascioliasis. In addition, when the cattle get older, their immunity against *Fasciola* might decrease [58]. However, studies found a higher prevalence rate of fascioliasis in young cattle than in adults [48, 59] and rationalised that older animals would develop acquired immunity that resulted in resistance [60].

The higher prevalence of *Schistosoma* and *Fasciola* found in the northern parts of Côte d’Ivoire compared to the South might be explained by the many small multipurpose dams built in the North in the 1970s and 1980s. Indeed, dams play a crucial role in maintaining the lifecycle of snail-borne infections, and some studies have shown the presence of intermediate snail hosts of *Schistosoma* and *Fasciola* in North Côte d’Ivoire [61, 62]. In Senegal, trematode infection increased after the construction of large dams across major rivers in the Senegal River basin [63]. The northern part of Côte d’Ivoire is also home to the largest livestock production in the country. The lower prevalence found in the central, southern and western regions could mean that there is a lower parasite pressure on animals compared to the North. The lowest prevalence of schistosomiasis in slaughtered cattle was observed in Duekoué, the western forested area of the country. Studies in humans in Western Côte d’Ivoire showed that *S. mansoni* is the predominant species [20, 64]. Unlike in cattle, the highest prevalence of fascioliasis in small ruminants was observed in sheep in the forested areas. However, the sample size was not large enough to draw firm conclusions.

The prevalence of *Schistosoma* in cattle was lower on farms and higher in slaughterhouses, while the opposite was observed for *Fasciola*. These observations should be interpreted with caution because the two populations of cattle differ in important ways, and the animals tested were not from the exact same places. Therefore, results from farms could not be compared to those from abattoirs.

The following considerations are offered for discussion. First, farm populations are more diverse in regards to age and include calves and young cattle that have had little time to be infected. Second, the cattle at slaughterhouses may be ill, their illness being the reason that they are brought for slaughter. Third, the prevalence of bovine schistosomiasis is often higher in wet season compared to the dry season [16]. As the farm sampling in this study was carried out in the rainy season in the North (August 2018), we expected the prevalence of schistosomiasis to be higher as seen in other investigations [65, 66]. Yet the prevalence was low there and the high prevalence of schistosomiasis was found in dry season (March 2018) in the abattoirs. In regards to the low schistosomiasis prevalences, sedimentation technique has been shown to have a low sensitivity, depending on the protocol. This may be due to factors that affect coprology in general, such as variation in the distribution of eggs within a single faeces specimen; daily fluctuations of faecal production and consistency in the host; and daily fluctuations related to oviposition patterns of the parasite [67–69]. The sensitivity is also influenced by the time samples are left to sediment. This is due to the rapid hatching of *Schistosoma* eggs, which may occur during the sedimentation process before the sediment is observed on the slide [70]. In fact, after exposure to water, *Schistosoma* eggs can hatch within 20 min [71]. In this study, the total time eggs were left to sediment in water was 45 min. It may also be because not all *Schistosoma* eggs are excreted in the faeces, many are left trapped the in tissue [52]. Lastly, the immune reaction of host organisms against schistosomes is not primarily directed towards the elimination of adult worms but rather towards the suppression of worm fecundity [72], also leading to reduced egg output. However, with post-mortem examination, adult flukes can be easily detected in the mesenteric vein or liver.

The higher prevalence of *Fasciola* observed in farm cattle might be due to the higher endemicity of *Fasciola* in the breeding areas. In addition, faecal examination appears to be more sensitive for *Fasciola* egg detection. Corroborating this finding, a study in South Ethiopia found a higher prevalence of *Fasciola* in farm cattle [73]. The higher prevalence of fascioliasis in this study could be due to the fact that sampling was done during the rainy season in the northern part of Côte d’Ivoire. In fact, fascioliasis prevalence is known to vary depending on the season, with an increase in prevalence during the wet season [41, 74, 75]. In contrast, an abattoir study in Southwestern Nigeria reported a higher prevalence of fascioliasis in the dry season compared to the rainy season [76]. To investigate further the epidemiology of trematode infections in livestock in Côte d’Ivoire, a seasonal study of both infections is needed.

Our study has some limitations. First, we did not count the eggs of parasites in the faeces of farm animals; we only determined the prevalence but not the intensity of infection. Second, the sample sizes at some abattoirs were small especially for goats and sheep; in addition, farms from central, southern and western parts of Côte d’Ivoire were less sampled than farms in the North, as livestock populations are mainly concentrated in the northern parts of Côte d’Ivoire. Third, we did not record the history of the treatments that might influence the prevalence of trematodes on farms. However, this study highlights the importance of veterinary trematodes in Côte d’Ivoire, as well as provides prevalence information for regions not previously sampled and updates the existing literature on livestock schistosomiasis and fascioliasis in West Africa, particularly in Côte d’Ivoire.

## Conclusions

Animal schistosomiasis and fascioliasis are prevalent in abattoirs and on farms across Côte d’Ivoire, with the highest prevalence found in the North. Our study revealed that *Fasciola* and *Schistosoma* infection are both associated with older age cattle and male sex. In view of our findings, a strategic control programme for cattle trematodes is warranted. An important component of such a control programme is to water cattle in troughs rather than to let them freely consume water from ponds, in order to reduce faecal contamination.

## Methods

### Study area

**Fig. 2:**
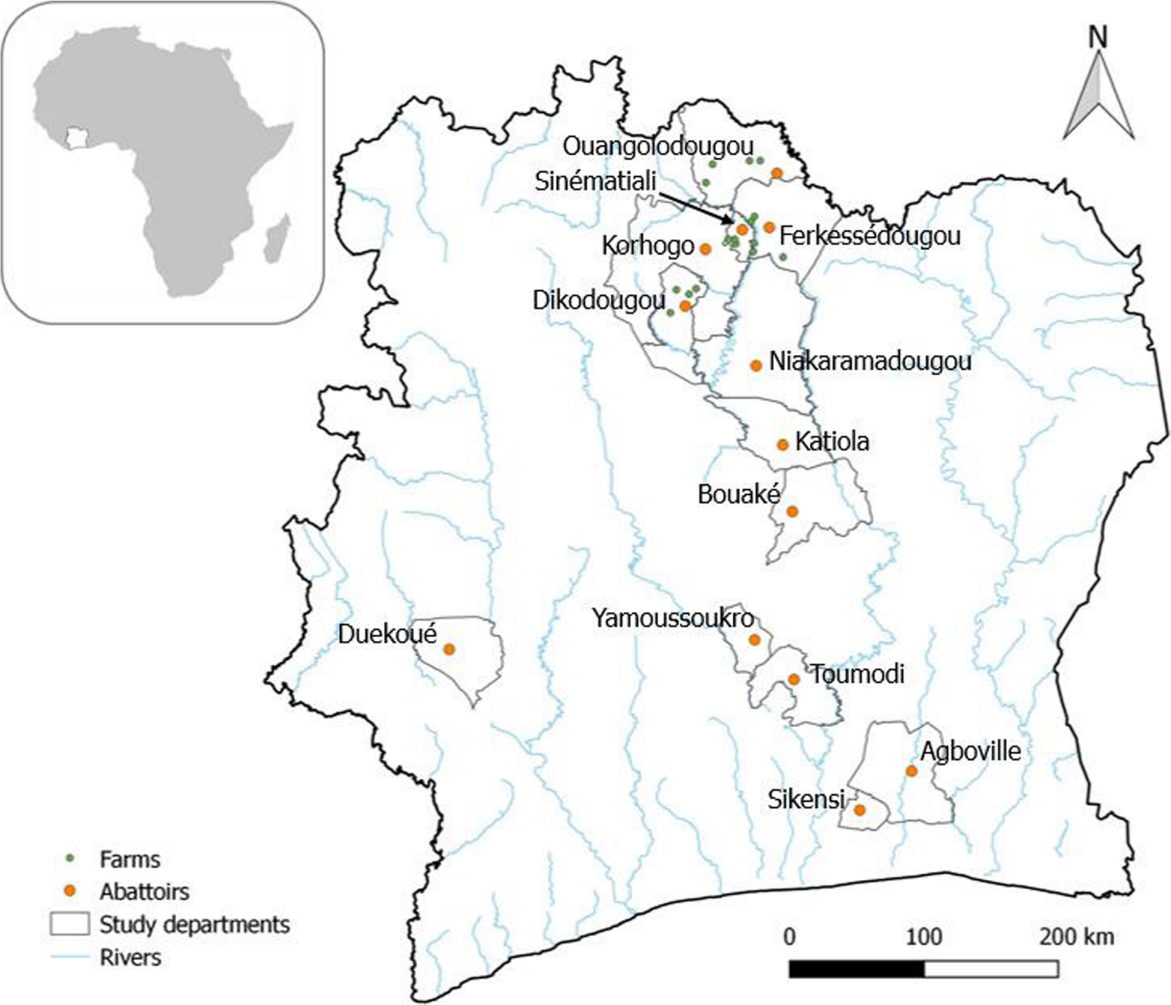
Map of Côte d’Ivoire highlighting the 13 departments where sampling for fascioliasis and schistosomiasis was conducted. Livestock from 11 abattoirs and 16 farms were sampled during two cross-sectional surveys in 2018. Software QGIS version 2.16.0 ‘Nødebo’ (QGIS Development Team) [43], was used to make the map

The study was conducted in 13 departments located in the northern, central, southern and western parts of Côte d’Ivoire: Ouangolodougou, Ferkessédougou, Sinématiali, Korhogo, Dikodougou, Niakaramadougou, Katiola, Bouaké, Yamoussoukro, Toumodi, Agboville, Sikensi and Duekoué (Fig. 2). The areas in the North and the Centre are characterised by a tropical climate and savannah vegetation with some forested areas. The South and West are in the tropical rainforest zone. Rainfall is higher in the southern and western parts, compared to the central and northern parts. The South and West also have less grass for livestock than the central and northern parts. The hydrography is composed of rivers running from North to South and several small multipurpose dams, mainly used for cattle breeding in the North [77]. The rivers and dams provide suitable habitats for intermediate host snails of schistosomiasis and fascioliasis [61] and the savannah with its grass is favourable for cattle breeding.

### Sampling and study design

Two cross-sectional surveys were carried out in March and August of 2018 to determine the prevalence of schistosomiasis and fascioliasis in cattle, goats and sheep. The first survey was conducted to obtain an overview of the prevalence of schistosomiasis and fascioliasis in slaughtered animals, in 11 of the 13 departments across the full geographical range. At least 3 days were spent at each local abattoir, where the livers and small intestines from slaughtered livestock were excised during the routine meat inspection. The samples were stored and transferred to a nearby laboratory of the “Direction Départementale du Ministère des Ressources Animales et Halieutiques”. There, livers and small intestines were examined for *Fasciola* and *Schistosoma* flukes. Demographic information such as sex, age, breed and origin were recorded. The animals in the abattoirs came from the local villages or in the northern part of the country, in particular for central, southern and western abattoirs. Of course, there were transhumant animals from the northern border countries, Mali and Burkina Faso, but these animals were not included in the study.

The second cross-sectional survey aimed to determine the prevalence of schistosomiasis and fascioliasis in farm animals and was restricted to the departments of Ouangolodougou, Ferkessédougou, Sinématiali and Dikodougou. These sites were chosen due to the high prevalences of infection detected there in the first cross-section study, in combination with the presence of dams and water bodies frequented by both humans and animals, in the area. A total of 16 farms (in villages) were randomly selected, four farms from each department. Rectal sampling was conducted under license as mentioned in the ethics approval and consent to participate section. After consent from the farmers, veterinary technicians collected faecal samples from the rectums of cattle, goats and sheep, as well as demographic information for each animal. Both abattoir and farm samplings were carried out in the dry season (March) and rainy season (August), respectively. The average monthly temperature and precipitation for the study months sampled are shown in the supplemental material (Additional file 1).

The sample size was determined based on the expected prevalence (*p*) of bovine fascioliasis and the desired absolute precision (*d*) and 95% confidence interval (CI) by using the formula *n* = (Z^2^ * *p* * (1-*p*)) / *d*^2^, where Z = 1.96, *p* = 60% and *d* = 5% [78]. Thus, a sample size of 369 animals was ascertained for each group of animals, from either slaughterhouses or farms.

### Laboratory procedures

**Fig. 3:**
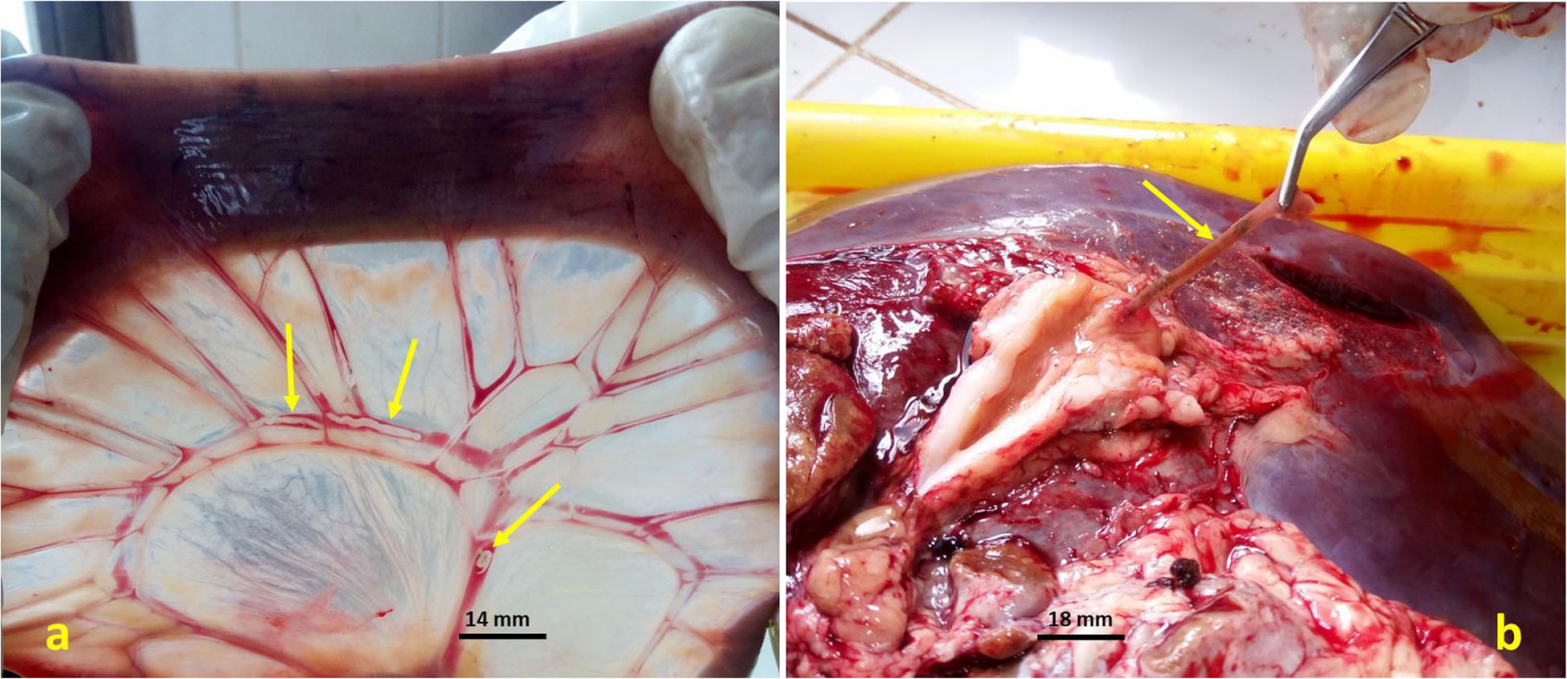
*Schistosoma* flukes in mesenteric veins of the small intestine (**a**) and a *Fasciola* fluke in a cattle liver (**b**)

Macroscopic inspection of the small intestine and liver of animals for adult flukes was carried out in the laboratory. The mesenteric veins of the small intestine (Fig. 3a) and hepatic veins were examined for schistosomes. *Fasciola* were recovered from the biliary duct and the parenchyma of the liver (Fig. 3b) [45].

Faecal samples from farm animals were processed by a sedimentation method [79]. In brief, approximately 5 g of faeces was placed in a cup and mixed with 30 ml of tap water. The faecal suspension was sieved through a 400 μm sieve into a 500 ml conical beaker. Tap water was added until the beaker was almost full. The conical beaker sat undisturbed for 15 min. The supernatant was decanted, and the sediment re-suspended in tap water. The process of suspension and decanting was repeated three times. Finally, using a pipette, a drop of the sediment was transferred onto a slide, stained with methylene blue (1%) and examined under a microscope for parasite eggs.

### Statistical analysis

Data were double entered into EpiInfo version 3.5.4 (Centers for Disease Control and Prevention; Atlanta, USA) and checked for internal consistency. R software version 3.5.2 [80] was used to calculate proportions and 95% CIs. Associations between parasitic infections and risk factors (sex, age and breed) were assessed using generalized estimating equation (GEE) analysis for binary outcomes with farms and slaughterhouses as clusters. Animals from Agboville, Sikensi and Duekoué were not included in the GEE analysis due to missing data on animal traits in these areas.

## Supplementary Information

**Additional file 1.** Climate diagram of the northern (a), central (b), southern (c) and western (d) areas of Côte d’Ivoire in 2018.

## Data Availability

The datasets used and/or analysed during the current study are available from Dr. Oliver Balmer on reasonable request.

## Abbreviations

CI: Confidence interval
OR: Odds ratio
*S*: *Schistosoma*
*F*: *Fasciola*
*p*: Prevalence
*d*: Desired absolute precision
GEE: Generalized estimating equation
