# Supplementary figures and images for: Prevalence and distribution of livestock schistosomiasis and fascioliasis in Côte d’Ivoire: results from a cross-sectional survey
Source: BMC Vet Res. 2020 Nov 17;16:446. doi: 10.1186/s12917-020-02667-y (PMC7672978; doi:10.1186/s12917-020-02667-y)

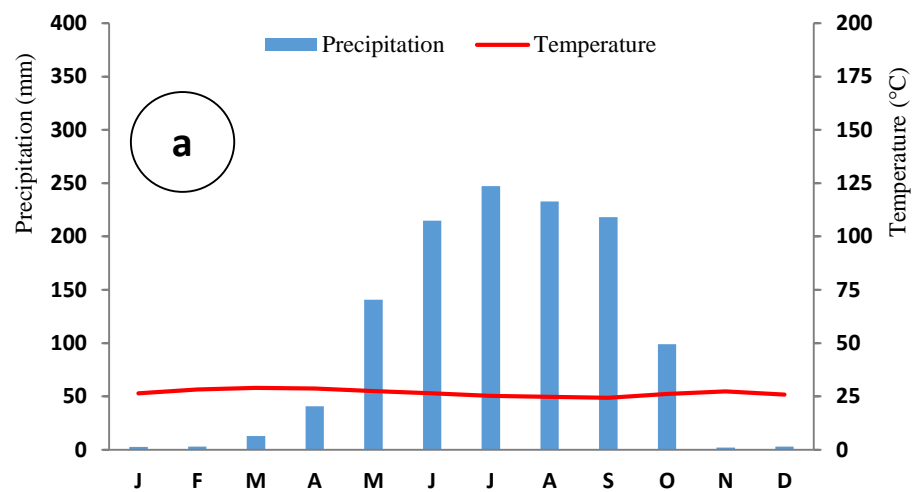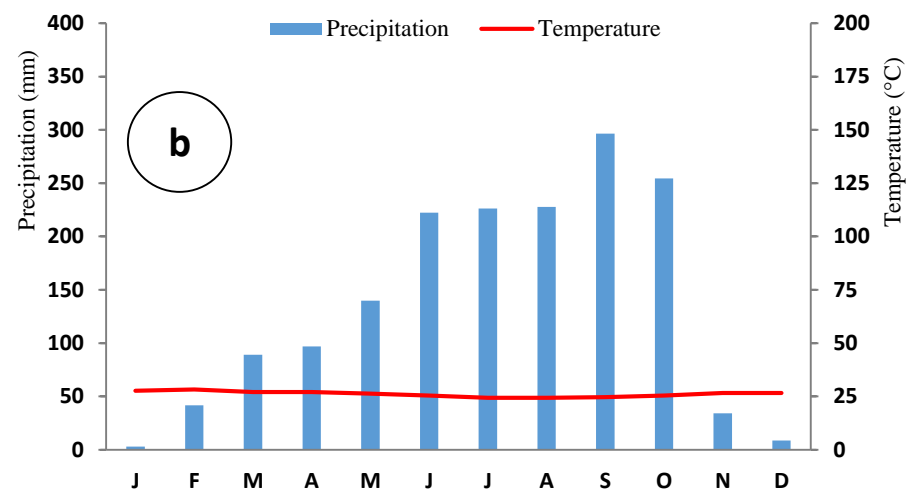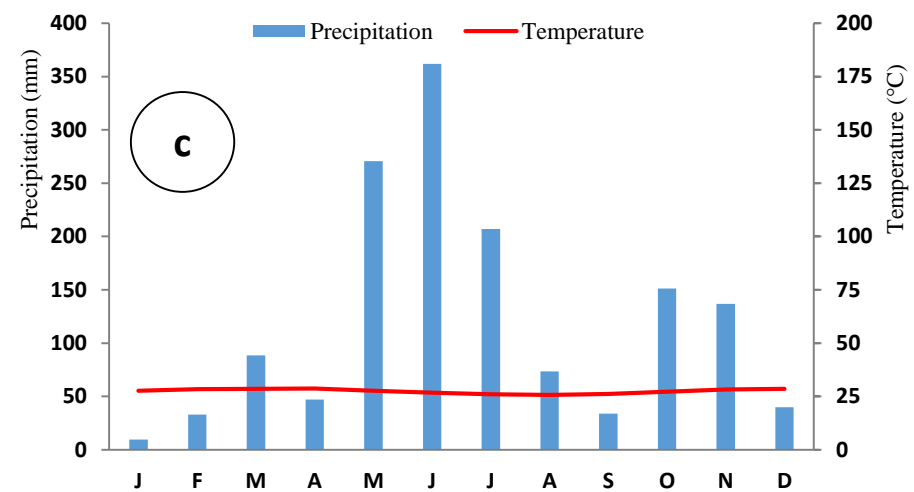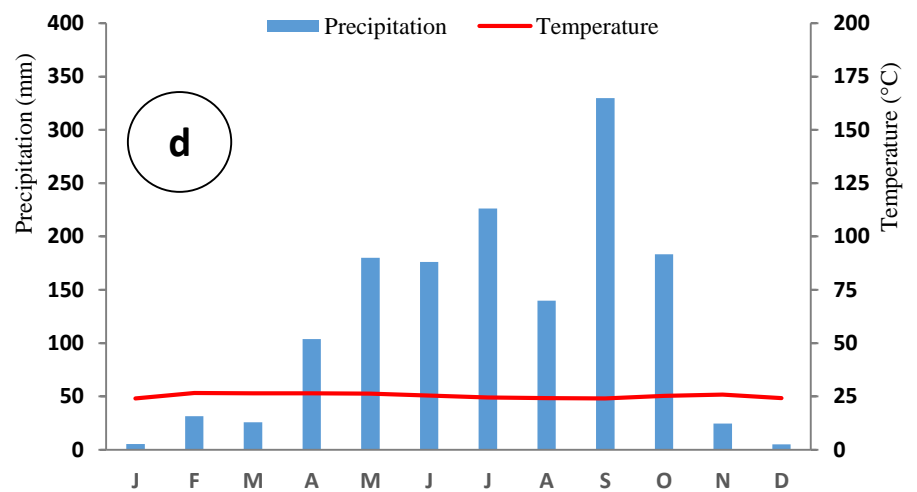

Supplement: Supplementary file 1 — Additional file 1. Climate diagram of the northern (a), central (b), southern (c) and western (d) areas of Côte d’Ivoire in 2018. [file 12917_2020_2667_MOESM1_ESM.pdf]
